# Reduced insulin/IGF-1 signalling upregulates two anti-viral immune pathways, decreases viral load and increases survival under viral infection in *C. elegans*

**DOI:** 10.1007/s11357-024-01147-7

**Published:** 2024-04-08

**Authors:** Elizabeth M. L. Duxbury, Hanne Carlsson, Annabel Kimberley, Yvonne Ridge, Katie Johnson, Alexei A. Maklakov

**Affiliations:** School of Biological Sciences, University of East Anglia, Norwich, UK

**Keywords:** Insulin signalling, Anti-viral immunity, Healthy aging, Orsay RNA virus, Rate-of-senescence

## Abstract

Reduced insulin/IGF-1 signalling (rIIS) improves survival across diverse taxa and there is a growing interest in its role in regulating immune function. Whilst rIIS can improve anti-bacterial resistance, the consequences for anti-viral immunity are yet to be systematically examined. Here, we show that rIIS in adult *Caenorhabditis elegans* increases the expression of key genes in two different anti-viral immunity pathways, whilst reducing viral load in old age, increasing survival and reducing rate-of-senescence under infection by naturally occurring positive-sense single-stranded RNA Orsay virus. We found that both *drh-1* in the anti-viral RNA interference (RNAi) pathway and *cde-1* in the terminal uridylation-based degradation of viral RNA pathway were upregulated in early adulthood under rIIS and increased anti-viral resistance was not associated with reproductive costs. Remarkably, rIIS increased anti-viral gene expression only in infected worms, potentially to curb the costs of constitutively upregulated immunity. RNA viruses are found across taxa from plants to mammals and we demonstrate a novel role for rIIS in regulating resistance to viral infection. We therefore highlight this evolutionarily conserved signalling pathway as a promising therapeutic target to improve anti-viral immunity.

**Supplementary Information:**

The online version contains supplementary material available at 10.1007/s11357-024-01147-7.

## Introduction

Promoting healthy ageing remains a global challenge in the face of viral pandemics [1]. Immunosenescence, the decline in immune function with age, leads to decreased pathogen resistance and increased mortality, and is almost universal across taxa [2–4]. Ageing is associated with the reduced production and efficiency of immune cells, increased inflammation, reduced responsiveness to vaccines and a decline in tolerance of infection [5]. Indeed, age is a major risk factor for most infectious diseases and reduced immune function has been linked with several age-associated diseases such as Alzheimer’s and cancer [6, 7]. Nevertheless, the relationship between ageing and immunity is complex and poorly understood [8].

Resource allocation theory proposes that whilst increased immunity improves survival in the face of infection, maintaining a strong immune response could be costly and trade-off with longevity in the absence of infection [9–13]. However, long-term experimental evolution of increased longevity, via selection for delayed reproduction, led to simultaneously reduced immune overactivation in older individuals and increased survival under infection in the fruit fly, *Drosophila melanogaster* [14]. This suggests that immunity and longevity are underpinned by shared molecular mechanisms and that age-specific application of lifespan-extension interventions could have a positive effect on immune response [8].

There is increasing interest within biogerontology and translational medicine fields in harnessing reduction in insulin/IGF-1 signalling (rIIS) to increase healthy lifespan. Nutrient-sensing IIS is evolutionarily conserved and shapes longevity and ageing [15, 16]. Experimentally downregulating IIS signalling via genetic, dietary or pharmacological interventions increases lifespan and/or improves health in studies across *Caenorhabditis elegans* nematodes, *D. melanogaster* fruit flies and mammals [17, 18]. In humans, a naturally occurring polymorphism in FOXO (FOXO3A genotype), a downstream component of both nutrient-sensing pathways, is strongly correlated with human longevity [19, 20]. Growing evidence suggests that nutrient-sensing pathways may regulate immune and life history responses to pathogens [12], yet research has mainly focused on anti-bacterial immunity, whilst relatively little is known about the possible role of IIS in anti-viral immunity [8].

rIIS may directly regulate resistance to viruses by targeting the expression of genes involved in anti-viral immunity. Indeed, the rIIS in *C. elegans daf-2* mutants enhances RNA interference (RNAi), which plays a key role in anti-viral defence and silencing of transposable elements [8, 21, 22], but it is not known whether rIIS improves survival under naturally occurring viral infection. Furthermore, high constitutive upregulation of RNAi can be costly, suggesting a potential trade-off between anti-viral immunity and longevity in the absence of viral infection [23]. Finally, *C. elegans* have another, recently discovered, anti-viral defence system—3′ terminal uridylation of viral RNAs [24]—and the role of rIIS in regulating this pathway is unknown.

Here, we asked whether rIIS can simultaneously improve anti-viral immunity and longevity without a cost to reproduction via one or both of these anti-viral defence systems, or if there would be life history trade-offs as predicted by resource allocation theory. Using the only known natural viral pathogen of *C. elegans*, the single-stranded RNA Orsay virus [25, 26], we tested the effect of adulthood-only rIIS, via *daf-2* RNAi, on the expression of key genes in two anti-viral defence mechanisms (*drh-1* for anti-viral RNAi and *cde-1* for 3′ terminal uridylation), whilst monitoring age-specific changes in viral load, survival and reproduction in *C. elegans*. We found that rIIS in adults was associated with the upregulation of both anti-viral immunity pathways, reduced viral load in old age and increased survival with no cost to reproduction in *C. elegans* under viral infection. Thus, our results suggest that rIIS is a promising therapeutic target to improve anti-viral resistance.

## Materials and methods

### Strain maintenance

*C. elegans* nematodes (JU2572 strain) that were stably infected with Orsay virus (JUv2572 isolate) were defrosted from stocks kindly provided by the lab of Prof. Marie-Anne Félix (IBENS-ENS, Paris, France). JU2572 were reared through two generations before setup, on nematode growth medium (NGM), supplemented with the fungicide nystatin and antibiotics streptomycin and ampicillin (each at 100 µg/mL, as standard recipes, e.g., [27]), and seeded with the antibiotic-resistant *Escherichia coli* bacterial strain OP50-1 (pUC4K, from J. Ewbank at the Centre d’Immunologie de Marseille-Luminy, France). Controlled 3 h egg lays from the grandparents and day 2 parents of experimental individuals were used to standardise parental age and we bleached eggs from half of the JU2572 grandparents, to remove viral infection [25]. All JU2572 populations and experimental individuals were kept at 20 °C.

### Knockdown of *daf-2* expression via RNA interference

To reduce adulthood insulin/IGF-1 signalling (rIIS), we fed late-L4 larvae with *E. coli* bacteria expressing double-stranded RNA for the insulin-like sensing receptor homolog, *daf-2*, which decreases mRNA levels of the transcribed *C. elegans daf-2* systemically [28, 29]. Previous work found that feeding RNAi downregulates *daf-2* expression by 50% in *C. elegans* on day 2 of adulthood [30]. RNase-III-deficient, IPTG-inducible HT115 *E. coli* bacteria with an empty plasmid vector (L4440) was used as the control and the L4440 plasmid had a *daf-2* insert for *daf-2* RNAi (as [29, 31, 32]). RNAi clones were acquired from the Vidal feeding library (Source BioScience, created by M. Vidal lab, Harvard Medical School, USA) and were verified via sequencing prior to delivery. All experimental individuals developed on the empty vector control. Experimental plates contained IPTG, ampicillin and nystatin (as standard recipe above).

### Survival and reproduction assays

To determine the effect of reduced IIS, via *daf-2* RNAi in adults, on age-specific survival and age-specific reproduction under Orsay virus (OrV) infection, we established four experimental treatments that were the full-factorial combinations of RNAi treatment (empty vector, ev or *daf-2* RNAi) and virus treatment (either lifelong virus infection from egg or no virus infection). Survival and reproduction assays were performed on the same experimental individuals for all treatments in parallel, across five time-staggered independent blocks, for logistical reasons and to capture any between-block variation. One experimental block had a very high rate of matricide (internal hatching of offspring within the parent that kills the parent) after the reproduction assay had completed, so it was excluded from the survival assay. Final sample sizes totalled across blocks for ev, *daf-2* RNAi, ev + virus and *daf-2* RNAi + virus treatments were *n* = 286, 291, 308 and 310 individuals/treatment respectively for survival and *n* = 174, 179, 210 and 210 individuals/treatment respectively for reproduction. The large sample sizes were chosen to maximise statistical power, as recommended for lifespan assays [33].

Individual age-synchronised, late-L4 OrV-infected or uninfected hermaphrodites were set up on separate plates, seeded with either ev or *daf-2* RNAi, that was grown to an ad libitum lawn, prior to the addition of 30 µL of antibiotic mix to inhibit growth of any contaminating bacteria (1:1 ratio of 100 µg/mL ampicillin, and 12.5 µg/mL tetracycline).

Age-specific offspring production (fecundity) was assayed over the first 6 days of adult life (the reproductive window for *C. elegans* hermaphrodites), by transferring all individuals to fresh plates daily, following the standard temporal resolution for *C. elegans* studies [31, 32, 34]. Plates were scored for viable adult offspring 2.5 days later. Total lifetime reproduction (lifetime reproductive success) was calculated per individual as the sum of age-specific offspring counts across the first 6 days of adulthood.

Survival was recorded daily for the entire lifespan for all experimental individuals. Worms were grouped as ten worms per plate and transferred to fresh plates every 2 days after the reproductive period. Mortalities were classed as death, matricide, death due to expulsion of internal tissue, or an accidental experimental loss (the last two categories were censored).

### Quantification of anti-viral gene expression, viral load and *daf-2* downregulation

We used quantitative reverse transcriptase polymerase chain reaction (qRT-PCR) to quantify the expression of *drh-1* (Dicer-related helicase 1), a key component of the *C. elegans* anti-viral RNAi pathway [24, 35], *cde-1* (co-suppression defective-1) which encodes the anti-viral terminal uridylation protein [24] and the Orsay virus RNA1 gene that encodes the viral RdRP protein required for viral replication, recommended as a reliable indicator of viral load [36]. The endogenous housekeeping gene *actin-3* was used as an internal control (as [37]). Anti-viral gene expression and viral load were assayed on day 2 and day 15 of adulthood to capture the start and middle of infection, in 15 pools of three individuals (to extract sufficient RNA) from each of the four life history assay treatments. Using identical methods, we also quantified the expression of *daf-2* (dauer formation- 2) in day 2 adults, to determine the extent of *daf-2* knockdown after 24 h on *daf-2* RNAi in the *C. elegans* JU2572 strain.

Using the Power SYBR™ Green Cells-to-CT™ Kit (Invitrogen, Cat#4,402,953), worms were lysed, and total mRNA was extracted with DNase I treatment to remove gDNA, reverse transcribed to cDNA and qRT-PCR with SYBR green, with all kit reagents and following established protocols for *C. elegans* [35, 38]. Primer sequences are listed in Table S6.

### Statistical analyses

All analyses were conducted in R version 4.3.1 [39]. Total lifetime reproduction data were plotted using the R package dabestr (data analysis using bootstrap-coupled estimation), for visualisation [40].

Age-specific survival was analysed using a Cox proportional hazards mixed-effect model, coxme [41], fitting RNAi, virus treatment and their interaction as fixed effects, with experimental block and plate ID as random effects. Separate analyses were conducted with matricides either censored or classed as deaths. The age at death response variable contained a coding variable to distinguish deaths from censored individuals. For all survival analyses, the *z*-scores and *p*-values were reported, with the *z*-score defined as the log of the hazard ratio (risk of death, exp(coef)) divided by the standard error of the log hazard ratio.

Age-specific mortality rates were estimated using Bayesian survival trajectory analysis in the BaSTA package (ver. 1.9.5, [42]). We compared ten models of actuarial ageing using Gompertz, Logistic and Weibull mortality functions, with either a simple, Makeham [43] or bathtub [44] shape, or an exponential model with simple shape (representing no senescence. Age-specific mortality curves were fitted from the first day of adulthood until death. Using the ‘multibasta’ function of the BaSTA package, we ran four parallel simulations of each model, for 150,000 iterations, with a burn-in of 15,001 iterations and a thinning of 150, allowing for robust convergence of all models. Models were compared using deviance information criteria (DIC values [45]. The Gompertz-Makeham model (Eq. 1) had the lowest DIC, so was selected as the most appropriate mortality rate function (Table S7). Mortality rate:

1 $${\mu }_{0}\left(x|b,c\right)\text{=}c+{e}^{{\text{b}}0+{\text{b1x}}}$$

The Makeham parameter (*c*) adds a constant that represents age-independent mortality to the Gompertz mortality function. Beta parameters describe baseline (b0) and exponential increase in mortality with age or rate-of-senescence (b1). To achieve low serial autocorrelation for all parameter estimates (< 5%), we ran the Gompertz-Makeham model for 1,000,000 iterations, with a burn-in of 100,001 iterations and thinning of 1000 (Table S2). The posterior distributions of mortality parameters all converged (Figure S3) and were compared between treatments using Kullback–Leibler divergence calibration (KLDC; Table S3). KLDC values closer to 0.5 indicated minimal difference between the distributions and values approaching 1 implied a larger difference [46, 47]. We considered a KLDC value > 0.85 to indicate a substantial difference between treatments, following established practice [48, 49].

Age-specific reproduction was analysed using generalised linear mixed-effects models to account for temporal pseudoreplication of repeated measures on the same individuals across age, with the template model builder package, glmmTMB [50]. Models were fitted with generalised Poisson or zero-inflated generalised Poisson error structure and were compared using AIC values of model fit with the AICtab function in the bbmle package, following Brooks et al. [51], to account for under- or over-dispersion and for zero-inflation. Age and a quadratic form of age (age^2^) were fitted as fixed effects in both the conditional and zero-inflation model formula, and significance assessed in each case. The age^2^ term controlled for the curved (nonlinear) trajectory of reproduction across age [52]. A maximal model was fitted with RNAi, virus treatment and their three-way interaction with age or age^2^ as fixed effects, and plate ID, block and experimenter as random effects. Stepwise model simplification was conducted [53] and the converging model with the best AIC fit was presented (as [51]). Total reproduction was analysed with a generalised linear mixed-effects model with RNAi, virus and their interaction as fixed effects, and block fitted as a random effect.

Relative gene expression was calculated as ΔCt, the difference between the qRT-PCR cycle thresholds (Ct values) of the target gene of interest (*drh-1*, *cde-1* or OrV RNA-1) and the reference gene, for each sample. The arithmetic mean of the Ct values for the two technical replicates per gene, per sample was used in ΔCt calculations. Fold change in gene expression (2^−ΔΔCT^) was calculated using mean ΔCt across all biological replicates per RNAi treatment [31].

Normalised anti-viral gene expression (2^−ΔCT^) was normally distributed (Shapiro–Wilk normality test) and analysed separately for *cde-1* and *drh-1* genes using a generalised linear model with Gaussian distribution, including a three-way interaction between day (day 2 or day 15 of adulthood), virus and RNAi treatments. Post hoc pairwise comparisons between treatments were made using the Welch two-sample *t*-test (to account for unequal variances). Expression plots were produced with the ggplot2 dotplot function with the standard dot density method for the *y*-axis, such that normalised gene expression values that fall within the default 1/30th of the range of the data for that treatment were binned along the *y*-axis for clarity but still represented as separate points. Binning has indistinguishable effect on the appearance of the plot and no data was binned for the analysis.

Viral load (normalised expression of OrV RNA-1 subunit) was analysed with a generalised linear model with Tweedie distribution to account for non-normality and zero-inflation, using the tweedie and statmod packages [54–56]. The Tweedie index parameter of 1.8 was calculated using maximum likelihood with the tweedie.profile function in the tweedie package, corresponding to a Poisson-gamma distribution for the continuous data that included a positive mass at zero. A two-way interaction between the fixed factors of RNAi treatment and day was fitted in the model for the virus infection treatment. Viral load for the no virus infection treatment was analysed separately using a Wilcoxon rank-sum exact test for completeness, due to convergence problems with the combined model, since all values were 0.

## Results

### Reduced adulthood insulin/IGF-1 signalling improves survival under viral infection

Orsay virus (OrV) primarily infects the intestine of *C. elegans* causing damage to cellular integrity and whilst not lethal, it can reduce lifetime progeny production and spreads solely via horizontal faecal-oral transmission[25, 35, 36]. The *C. elegans*-specific OrV is closely related to the Nodaviridae family of RNA viruses that infect fish, invertebrates such as *Drosophila*, and mammalian cells (Flock House virus), so is of wider evolutionary and commercial significance [26].

To test the consequences of reduced IIS on survival under lifelong viral infection, we used the *C. elegans* JU2572 strain that had stronger intestinal symptoms to potent OrV isolate JUv2572 infection, yet the life history consequences and mechanisms of susceptibility are unknown [26, 57]. We reduced IIS, via feeding *daf-2* RNAi to *C. elegans* from the start of adulthood onwards. Since insulin signalling is crucial for development, growth and reproduction, downregulating these pathways in early life is usually detrimental to fitness; however, adulthood-only *daf-2* RNAi robustly doubles lifespan and has no cost to reproduction, across a range of environments in the absence of infection [30–32, 38].

**Fig. 1:**
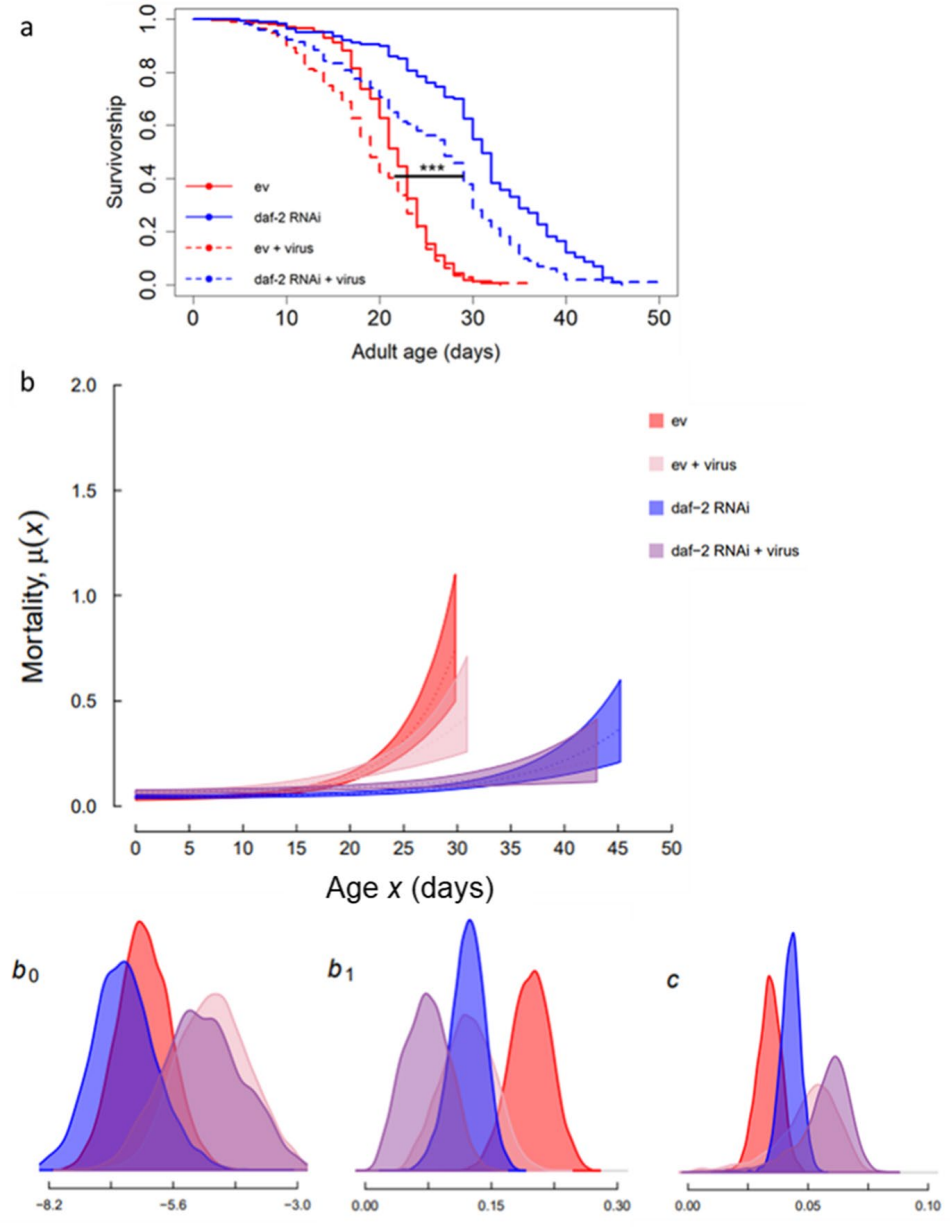
Reduced adulthood IIS, via *daf-2* RNAi, improves survival under viral infection. (**a**) Age-specific survival expressed as survival probability, (**b**) mortality curves with 95% confidence intervals as fitted by the Gompertz-Makeham model and posterior distributions for the three model parameters (b0: baseline mortality, b1: rate-of-senescence and c: age-independent mortality). Data was combined from four experimental blocks (total individuals per treatment: 286, 291, 308, 310; order as legend), with matricide censored. *C. elegans* nematodes (JU2572 strain) were infected with Orsay virus (potent JUv2572 isolate) from egg stage, or eggs were bleached to remove viral infection. All nematodes were maintained on empty vector (ev) *E. coli* bacteria during development and then either *daf-2* RNAi or ev during adulthood

We found that whilst lifelong Orsay virus infection was costly to JU2572 survival by increasing both baseline and age-independent mortality, reduced IIS in adulthood significantly improved both median and maximum lifespan under viral infection relative to infected individuals on empty vector (ev) control, by specifically reducing the rate-of-senescence (median, maximum lifespan *daf-2* RNAi + virus: 26, 50 days versus ev + virus: 19, 36 days; Cox proportional hazards mixed-effects model, coxme with matricides censored, RNAi: *z* = 4.40, *p* < 0.001; virus: *z* = 10.93, *p* < 0.001; RNAi x virus: *z* =  − 2.22, *p* = 0.027; Fig. 1; Fig. S1; Tables S1–S3).

Although virally infected individuals on *daf-2* RNAi suffered a survival cost of infection relative to uninfected individuals on *daf-2* RNAi, via increased baseline and age-independent mortality (median lifespan 26 days versus 31 days; coxme, virus: *z* = 4.11, *p* < 0.001; Fig. 1; Fig. S1, Tables S1–3), they still lived significantly longer than both infected and uninfected worms without reduced adulthood insulin signalling (median lifespan, infected ev: 19 days, uninfected ev: 22 days; coxme, *daf-2* RNAi virus versus ev virus, RNAi: *z* = 5.97, *p* < 0.001; *daf-2* RNAi virus versus ev no virus, RNAi: *z* = 6.93, *p* < 0.001).

Curiously, there was more death due to matricide (internal hatching of offspring within the parent) under viral infection or on *daf-2* RNAi (percentage of deaths due to matricide: 34% ev, 51% *daf-2* RNAi, 43% ev + virus and 51% *daf-2* RNAi + virus; Fig. S2). Increased matricide may have provisioned offspring with more resources, potentially improving their fitness, although we did not measure it here. Previous work showed that *C. elegans* offspring from *daf-2* RNAi-treated parents have higher fitness and even under non-pathogenic stress [32, 38], but the role of matricide is currently unclear. Despite this, *daf-2* RNAi still significantly improved the survival of individuals that did not matricide under viral infection relative to virally infected ev controls (coxme with matricide as dead, RNAi: *z* = 0.39, *p* < 0.001, virus: *z* = 4.54, *p* < 0.001; RNAi x virus: *z* = 0.71, *p* = 0.480; Fig. S2; Table S1).

### Reduced IIS does not affect reproduction under viral infection

**Fig. 2:**
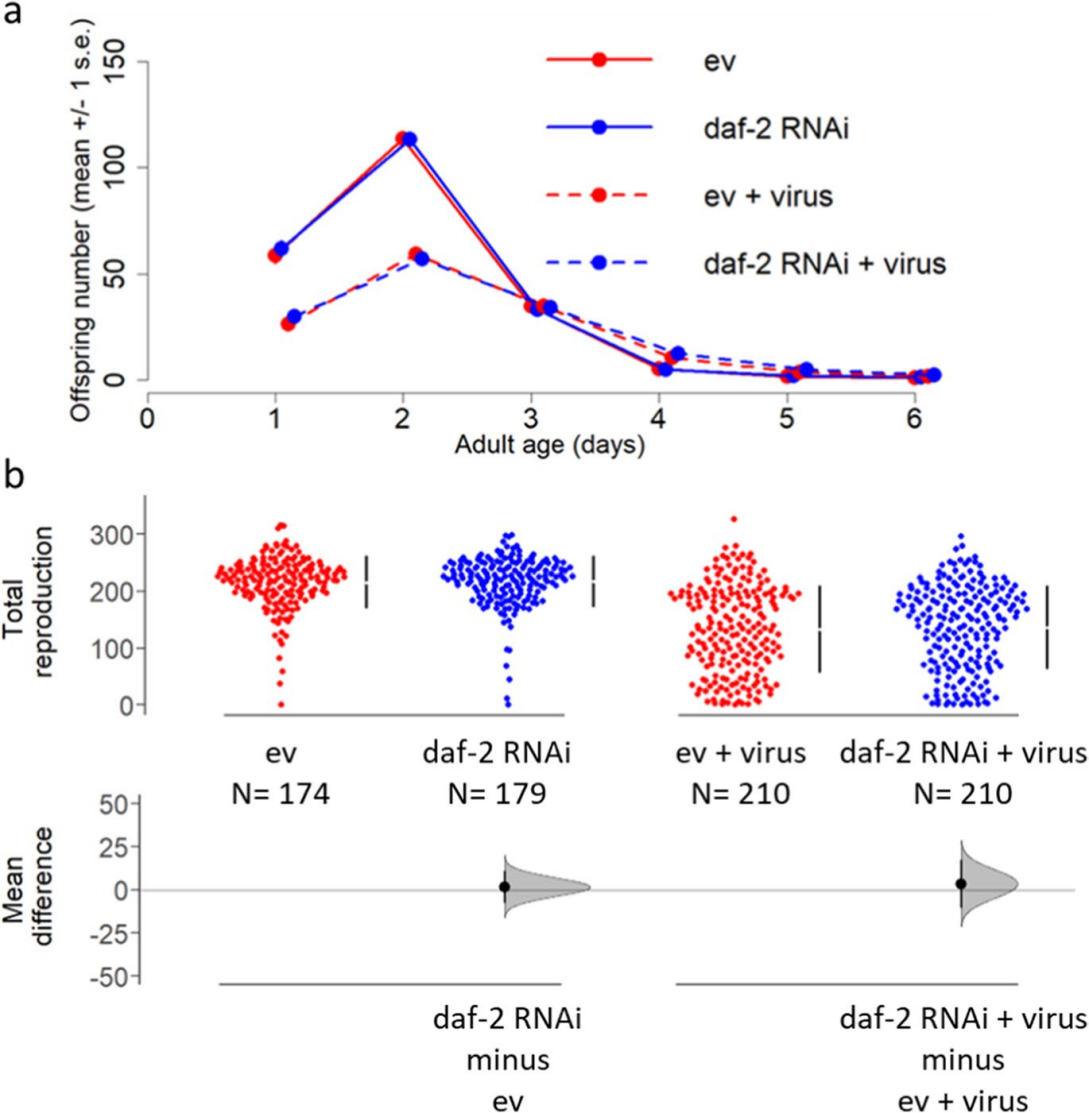
Reduced adulthood IIS, via *daf-2* RNAi, does not affect reproduction under viral infection. (**a**) Age-specific and (**b**) total lifetime reproduction data from five experimental blocks (*n* = 174, 179, 210, 210 individuals per treatment, order as legend). *C. elegans*, JU2572 strain, were infected with Orsay virus (JUv2572) from egg stage, or eggs were bleached to remove viral infection. All nematodes were maintained on empty vector (ev) during development and then either *daf-2* RNAi (‘daf-2’) or ev during adulthood. Mean daily offspring count ± 1 standard error (overlapping with data point dots as SE was very small) shown in (**a**). Total lifetime reproduction per individual was the sum of their age-specific reproduction counts. Mean difference between pairwise treatment comparisons of total reproduction (effect size) and 95% confidence intervals shown in (**b**) were derived using nonparametric bootstrap resampling in ‘dabestr’ R package [40]

To determine whether the increased lifespan and lowered rate-of-senescence under reduced adulthood insulin signalling were associated with a cost to reproduction under viral infection, we measured age-specific and total lifetime reproduction in the same individuals that were assayed for survival. Orsay virus infection significantly reduced both early reproduction (day 1 and day 2 of adulthood) and total lifetime reproduction, regardless of whether insulin signalling was reduced or at normal levels (zero-inflated generalised Poisson, ZIGP for age-specific, virus x age: *z* = 17.990, *p* < 0.001; virus x RNAi: *z* =  − 0.541, *p* = 0.588; ZI varied with age: *z* = 12.490, *p* < 0.001 and age^2^: *z* =  − 10.61, *p* < 0.001; Fig. 2a; Table S4; generalised linear mixed-effects model, GLMER for total reproduction, virus: *t* =  − 13.141, *p* < 0.001; RNAi: *t* =  − 0.222, *p* = 0.825; virus x RNAi: *t* =  − 0.220, *p* = 0.826; Fig. 2b; Table S5). Whilst there was no benefit to reduced adulthood IIS for reproduction, there was also no cost in the presence or absence of viral infection, contrary to the hypothesis of a trade-off between survival and reproduction under viral infection.

### Reduced adulthood IIS upregulates two anti-viral immune pathways in early adulthood and decreases viral load

Anti-viral immunity in *C. elegans*, other invertebrates and plants is based on the RNA interference pathway [58, 59]. Recent work suggests that mammals may also use RNAi-based innate immunity, although it is still under debate [8, 60]. A key component of the *C. elegans* anti-viral RNAi pathway is the viral recognition protein, Dicer-related helicase (DRH)-1, that recognises and targets replicating double-stranded OrV RNA for downstream silencing, reducing viral replication [24, 35]. DRH-1 is a homolog of the mammalian viral recognition protein RIG-1 that also functions in viral recognition and triggers downstream interference with viral replication via interferons [61, 62], recently being linked with SARS-CoV-2 detection in human lung cells [63, 64]. Anti-viral RNAi pathways in *C. elegans* are distinct from those triggered by exogeneous dsRNA [36]. A second, independent anti-viral innate immune pathway in *C. elegans* was recently discovered to operate in parallel to anti-viral RNAi, via a process of terminal uridylation [24]. The CDE-1 (co-suppression defective-1) protein marks the 3′ end of single-stranded viral RNA for degradation and is the homolog of mammalian TUT-4 and TUT-7 proteins [24].

To determine the possible role of insulin signalling in regulating these two anti-viral immune pathways in *C. elegans*, we tested the effect of reduced adulthood IIS, via *daf-2* RNAi, on the expression of the two key genes in these pathways, *drh-1* and *cde-1*, in early (day 2) and mid (day 15) adulthood in the presence or absence of viral infection. Since we hypothesised that reduced IIS could affect both immune response and tolerance to viral infection, we also assayed viral load (by measuring ORV RNA-1 levels) at these same timepoints. If rIIS solely affected tolerance, then we would expect no change in viral load.

**Fig. 3:**
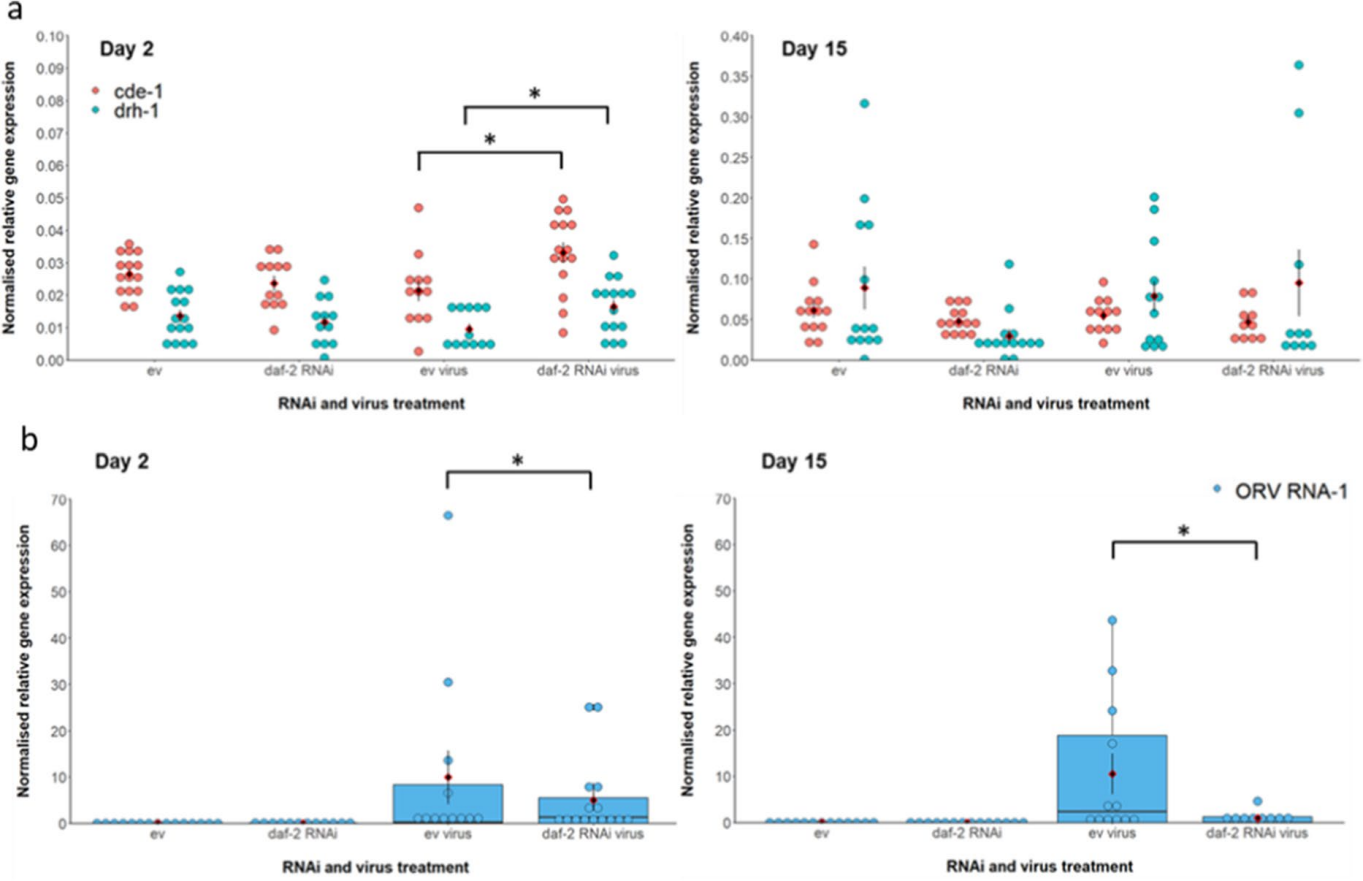
(**a**) Normalised anti-viral gene (*cde-1*, *drh-1*) expression and (**b**) viral load (*ORV RNA-1*) following *daf-2* RNAi treatment versus empty vector (ev) controls, in the presence or absence of lifelong Orsay virus infection in *C. elegans*. Orsay virus infection began from egg stage or eggs were bleached to remove viral infection. Adulthood RNAi (either daf-2 RNAi or empty vector, ‘ev’) is indicated. Gene expression was quantified in day 2 and day 15 adults using qRT-PCR. Arithmetic mean of biological replicates per treatment shown as a red diamond with ± 1 standard error bars. Boxplots for viral load indicate the median and interquartile range. Day 2 sample sizes of *n* = 15, 12, 12 and 15 pools of 3 worms per treatment respectively, and day 15 sample sizes of *n* = 13, 14 and 12 pools (except ‘daf-2 RNAi virus’ which had *n* = 9 for viral load), shown as separate points. Normalised gene expression (2^−ΔCT^) was calculated relative to expression of the *actin-3* reference gene [65]. Note the different *y*-axis scales for expression plots. Welch’s two-sample *t*-test was used post hoc to test for significance of pairwise treatment comparisons (*p < 0.05) for anti-viral gene expression and a generalised linear model with Tweedie distribution (index parameter = 1.8) was used for viral load data to satisfy zero-inflation and non-normality

Reduced IIS in adulthood, via *daf-2* RNAi, was associated with a significant upregulation in the expression of both *cde-1* by 68% and *drh-1* by 73% under viral infection on day 2 of adulthood, relative to infected ev controls (Welch’s two-sample *t*-test, *cde-1*, RNAi: *t* =  − 2.607, df = 24, *p* = 0.0153; *drh-1*, RNAi: *t* =  − 2.560, df = 24, *p* = 0.0171; Fig. 3a). Viral load was significantly reduced on both day 2 and day 15 of adulthood under *daf-2* RNAi relative to virally infected controls (generalised linear model, GLM, with Tweedie distribution, index parameter 1.8, RNAi: *t* = 2.469, *p* = 0.0174; day: *t* =  − 1.079, *p* = 0.286; RNAi x day: *t* = 1.600, *p* = 0.117; Fig. 3b). Day 2 of adulthood was before the divergence of survival trajectories between viral or *daf-2* RNAi treatments (Fig. 1a) and yet both immune pathways were upregulated under rIIS at this timepoint. In the absence of infection, neither *cde-1* nor *drh-1* was upregulated under *daf-2* RNAi on either day 2 (Welch’s *t*-test, *cde-1*, RNAi: *t* = 1.040, df = 21, *p* = 0.311; *drh-1*, RNAi: *t* = 0.687, df = 24, *p* = 0.499) or day 15 (Welch’s *t*-test, *cde-1*, RNAi: *t* = 1.325, df = 18, *p* = 0.202; *drh-1*, RNAi: *t* = 2.144, df = 14, *p* = 0.500; Fig. 3a), perhaps indicative of a cost of immune upregulation in the absence of infection.

On day 15 of adulthood, a timepoint at which there was reduced survival under viral infection but improved survival on *daf-2* RNAi (Fig. 1a), *cde-1* and *drh-1* expression in infected individuals was no longer significantly upregulated under rIIS relative to controls (Welch’s* t*-test, *cde-1*, RNAi: *t* = 0.841, df = 19, *p* = 0.411; *drh-1*, RNAi: *t* =  − 0.358, df = 13, *p* = 0.726), but viral load was still significantly higher in infected controls than in those on *daf-2* RNAi (Fig. 3). This suggests that early adulthood changes in anti-viral gene expression under rIIS were beneficial in reducing viral load both earlier and later in the course of infection and that reduced IIS may also have improved tolerance to viral infection even in the absence of upregulated *cde-1* and *drh-1* at day 15. Curiously, virally infected worms on the empty vector control did not significantly upregulate either *cde-1* or *drh-1* at either day 2 or day 15, relative to uninfected controls (GLM, *cde-1*, virus: *t* =  − 0.667, *p* = 0.508; day: *t* = 4.535, *p* < 0.001; virus x day: *t* =  − 0.102, *p* = 0.919; *drh-1*, virus: *t* =  − 0.181, day: *t* = 3.460, *p* = 0.00114; virus x day: *t* =  − 0.189, *p* = 0.851; Fig. 3a), which likely contributed to their significantly reduced survival under viral infection (Fig. 1).

### Adulthood *daf-2* RNAi reduces *daf-2* expression in virally infected and non-infected JU2572

**Fig. 4:**
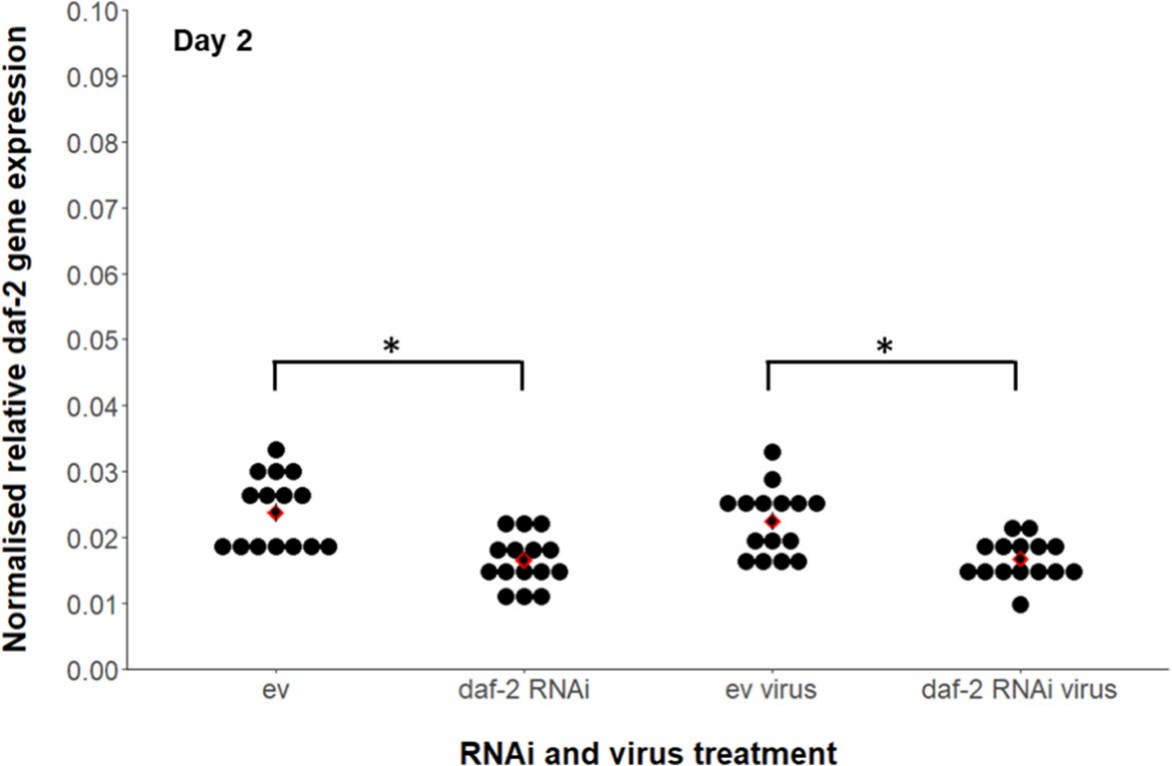
Normalised *daf-2* expression following adulthood *daf-2* RNAi treatment versus empty vector (ev) controls, in the presence or absence of lifelong Orsay virus infection in *C. elegans*. Gene expression was quantified in day 2 adults using qRT-PCR. Arithmetic mean of biological replicates per treatment shown as a red diamond with ± 1 standard error bars. Sample sizes of 15 pools of 3 worms per treatment. Normalised *daf-2* expression (2^−ΔCT^) was calculated relative to expression of the *actin-3* reference gene [65]. Welch’s two-sample *t*-test was used post hoc to test for significance of pairwise treatment comparisons (*p < 0.05) for anti-viral gene expression

To determine whether the *daf-2* RNAi treatment was variably effective in reducing *daf-2* expression in the JU2572 strain across virally infected and non-infected worms, we quantified *daf-2* expression in day 2 adults. Under adulthood *daf-2* RNAi, *daf-2* expression was downregulated by 25% in virally infected worms and by 30% in non-infected worms (based on mean gene expression fold change, 2^−ΔΔCT^). This represented a significant downregulation in the relative gene expression of *daf-2* by *daf-2* RNAi (Fig. 4; Welch’s *t*-test, virus-infected, RNAi: *t* =  − 3.635, df = 24, *p* = 0.001; non-infected, RNAi: *t* =  − 3.993, df = 24, *p* < 0.001). There was no significant difference in the extent of *daf-2* downregulation between virally infected and non-infected JU2572 (GLM, virus x RNAi: *t* =  − 0.627, *p* = 0.533; virus: *t* = 0.109, *p* = 0.914; RNAi: *t* = 4.260, *p* < 0.001). The absence of a significant change in anti-viral gene expression on *daf-2* RNAi in non-infected worms (Fig. 3) was therefore not due to variation in the efficiency of *daf-2* knockdown by *daf-2* RNAi. Similarly, since variation in *daf-2* expression was low between individuals within treatments (Fig. 4), the between-individual variability in anti-viral gene expression and viral load within *daf-2* RNAi treatments (Fig. 3) is not likely due to differences in the extent of *daf-2* downregulation, but rather reflecting between-individual differences in immune response.

## Discussion

In this study, we demonstrate for the first time to our knowledge that reduced IIS simultaneously upregulates two key anti-viral defence systems in *C. elegans*, decreases viral load and improves survival under viral infection, with no cost to reproduction. We further show that *daf-2* RNAi knockdown increases survival by reducing demographic rate-of-senescence.

Our work suggests that IIS regulates both longevity and anti-viral immunity through mechanisms that differ at least in part from the increasingly studied role of IIS in anti-bacterial resistance [66–69]. We demonstrate that the increased survival under viral infection when insulin signalling was reduced was associated with the upregulation of both anti-viral RNAi and terminal uridylation immune pathways in *C. elegans* in early adulthood, supporting the view that these pathways work in parallel [24], but highlighting a novel role for IIS in their regulation. Early adulthood upregulation of both *cde-1* and *drh-1* in infected individuals under rIIS was sufficient for improved survival and reduced viral load (as assayed by measuring ORV RNA-1 levels) later in adulthood, which agrees with previous work in the *C. elegans*-OrV system that found *cde-1* mutants had an earlier onset of infection and *drh-1* mutants had an increased persistence of viral infection [70].

Downregulation of IIS is known to improve organismal resistance to a variety of abiotic stresses such as heat and oxidative stress [71], so rIIS could additionally improve tolerance to viral infection [72], in addition to upregulated immunity and associated reduction in viral load. We note however that viral load was reduced in *daf-2* RNAi worms at day 15, suggesting that reduction in load is directly associated with improved survival in old age. Furthermore, since OrV is transmitted via feeding [25], perhaps behavioural avoidance of viral exudates, infected worms or even the bacterial food, together with variation in tolerance and immune activation, may explain some of the between-individual heterogeneity in viral load that we and others have observed [57, 70]. Whilst one study found no effect of OrV on *C. elegans* feeding rate when measured using fluorescent bead intake [73] , it remains to be tested whether OrV infection affects feeding rate in the more susceptible JU2572 strain and the possible consequences for survival or reproduction.

Under adulthood-only rIIS, we found that improved survival under viral infection did not trade-off with a cost to reproduction, despite the increased investment in immunity. By restricting rIIS to adulthood, previous work in *C. elegans* also extended lifespan without reproductive costs [31], including under different environmental stresses such as fluctuating temperature, visible light or UV irradiation [30, 38]. This is contrary to the trade-offs between immunity and reproduction or growth as seen in plants [74], insects [75] and vertebrates [76], and suggests that post-development reduction of insulin signalling may be key for cost-free anti-viral immunity.

The surprising observation that the *C. elegans* JU2572 strain did not, on average, significantly upregulate either *cde-1* or *drh-1* on day 2 or day 15 of adulthood under viral infection when IIS was not reduced, likely contributed to their reduced survival. This was similar to the degree of survival cost seen in the OrV-infected *drh-1* mutant in previous work [35], but unlike the absence of a survival cost from OrV infection seen in the *C. elegans* JU1580 strain that suffers fewer intestinal symptoms of infection than JU2572, despite having deficient *drh-1* [35, 57]. Since we observed that reproduction was also reduced under infection in JU2572, the lack of anti-viral immune upregulation does not appear to be due to adaptive resource reallocation. Whilst the mechanisms are currently unclear, our results suggest that anti-viral gene expression was ‘reactivated’ via the downregulation of IIS.

Consistent with theory that proposes it may be adaptive to downregulate immune signalling to conserve resources in the absence of infection [12], we found no upregulation of either anti-viral immune pathway in uninfected individuals, even under rIIS. Curiously, the *drh-1* deletion is widespread in *C. elegans* populations even though it increases viral susceptibility although it does not impair fitness in the absence of infection [35]. Furthermore, constitutive upregulation of RNAi can be costly [23]. Life history trade-offs therefore may constrain *drh-1* expression, to optimise costly investment in immune response, and can explain why we also found that anti-viral immunity was not upregulated in the absence of infection.

Previous work revealed additional mechanisms involved in the transcriptional response of *C. elegans* to OrV infection, such as the general intracellular pathogen response (IPR) that acts downstream of DRH-1 to promote anti-viral immunity [73, 77] and the STA-1 transcription factor that repressed IPR and anti-viral immune response but maintained normal lifespan in the absence of OrV infection, acting independently of anti-viral RNAi [78]. *C. elegans* STA-1 is a homolog of STAT that plays a key role in mammalian anti-viral innate immunity via JAK/STAT signalling [78]. Since anti-viral transcriptional response is likely to be influenced by *C. elegans* genetic background, age and duration of viral infection prior to gene expression assay, and these factors all varied between the studies, just as we observed differences in anti-viral gene expression between day 2 and day 15 of adulthood; it would be informative to test the effect of rIIS on IPR and *sta-1* expression under OrV infection of JU2572 *C. elegans* in future work.

Our results highlight rIIS as a promising target to simultaneously improve anti-viral immune function and survival, whilst slowing the rate-of-senescence under viral infection, with ultimate relevance for human immunosenescence and healthy ageing. RNA viruses are a concern for human health and food security. Notably, the Nodavirus family to which OrV is closely related [26] can cause large commercial losses in aquaculture. Since innate immunity and nutrient-sensing IIS pathway are evolutionarily conserved [8, 15, 16] and inhibition of several proteins in the IIS in mammals has also been associated with viral replication [79–81], we encourage future empirical work into the effects of rIIS on anti-viral immunity across species.

An important next step would be to explore the impact of non-genetic interventions that can reduce IIS on anti-viral immunity, since they may be more readily translated to humans. Dietary restriction (DR) is a powerful intervention that extends lifespan across diverse taxa [17]. Whilst reproductive costs of DR are common, they perhaps could be reduced via time-restricted fasting [82] that appears to operate at least partly via IIS in *C. elegans* [83]. Some evidence suggests that DR can improve the immune system and reduce susceptibility to infection [84, 85], and there is growing interest in the use of nutritional or pharmaceutical interventions for immunosenescence [86, 87].

In conclusion, our results suggest that downregulation of insulin signalling in *C. elegans* is a promising strategy to boost anti-viral defence and improve survival under viral infection, without reproductive costs, in addition to its beneficial role in anti-bacterial resistance and healthy ageing.

## Supplementary Information

Below is the link to the electronic supplementary material.

Supplementary file1 (DOCX 423 KB)

## Data Availability

The raw data are available at 10.6084/m9.figshare.24132663. The code for analyses are available at https://github.com/ElizabethMLDuxbury/Code-2023.git.
