# Supplementary material for: Reduced insulin/IGF-1 signalling upregulates two anti-viral immune pathways, decreases viral load and increases survival under viral infection in C. elegans
Source: GeroScience. 2024 Apr 8;46(6):5767–80. doi: 10.1007/s11357-024-01147-7 (PMC11493891; doi:10.1007/s11357-024-01147-7)
Supplement: Supplementary file 1 — Supplementary file1 (DOCX 423 KB) [file 11357_2024_1147_MOESM1_ESM.docx]

Reduced insulin/IGF-1 signalling upregulates anti-viral immunity upon infection resulting in decreased viral load and increased survival in old age

Elizabeth M. L. Duxbury^1^*, Hanne Carlsson^1^, Annabel Kimberley^1^, Yvonne Ridge^1^, Katie Johnson^1^ and Alexei A. Maklakov^1^

^1^School of Biological Sciences, University of East Anglia, Norwich, United Kingdom

^*^ [E.Duxbury@uea.ac.uk](mailto:E.Duxbury@uea.ac.uk) ; ORCID: 0000-0002-5733-3645

Short title: Insulin signalling and anti-viral resistance

**Supplementary Materials**

**Supplementary Figures**

**
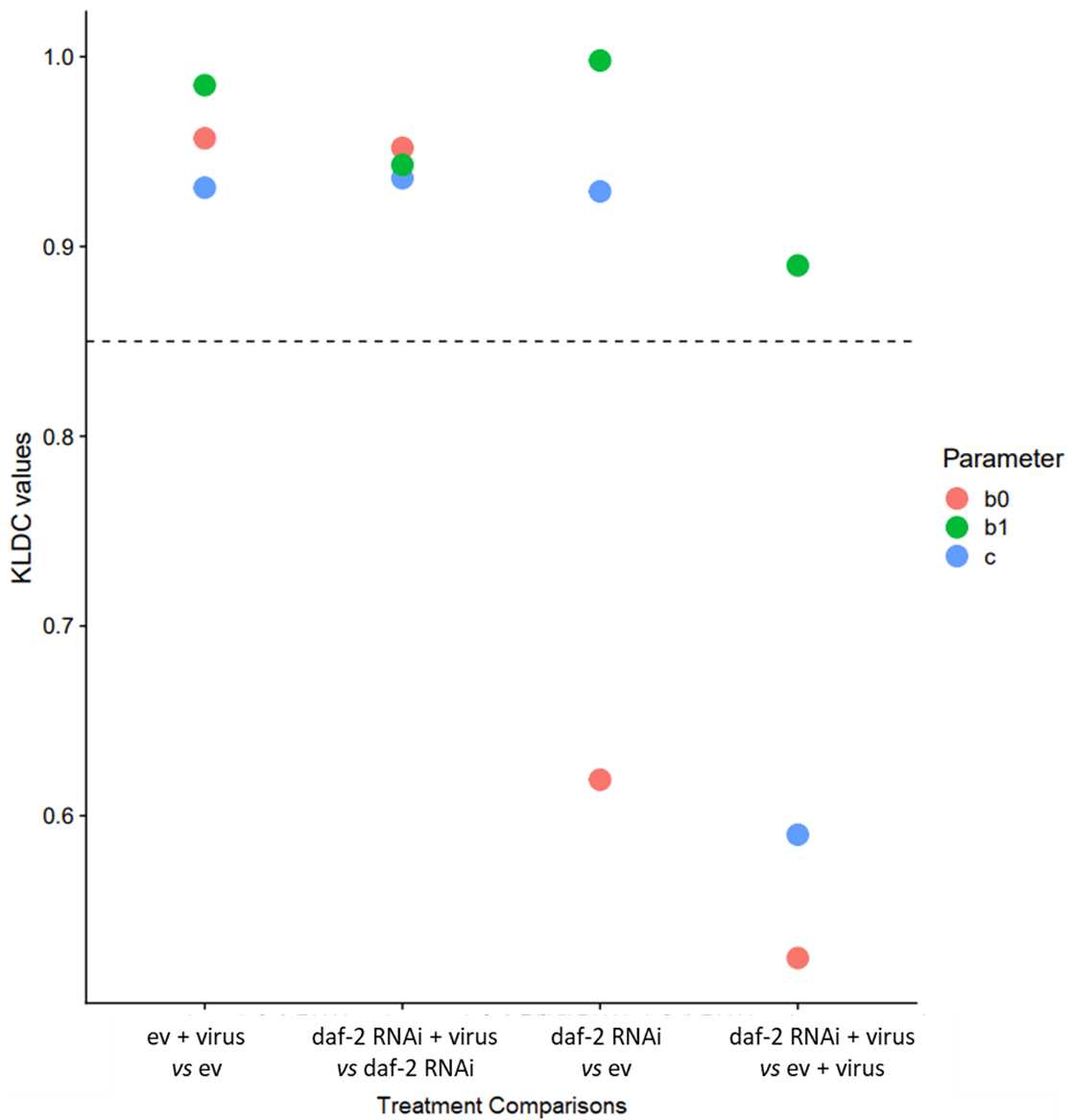
**

**Figure S1. Kullback-Leibler divergence calibration (KLDC) values for the comparisons of Gompertz Makeham mortality parameters between RNAi and virus treatments.** KLDC values which are above the 0.85 threshold (dotted line) indicate parameters with a substantial difference between the two treatments. The parameter b0 indicates baseline mortality, b1 is rate-of-senescence and c is the Makeham parameter of age-independent mortality.

**
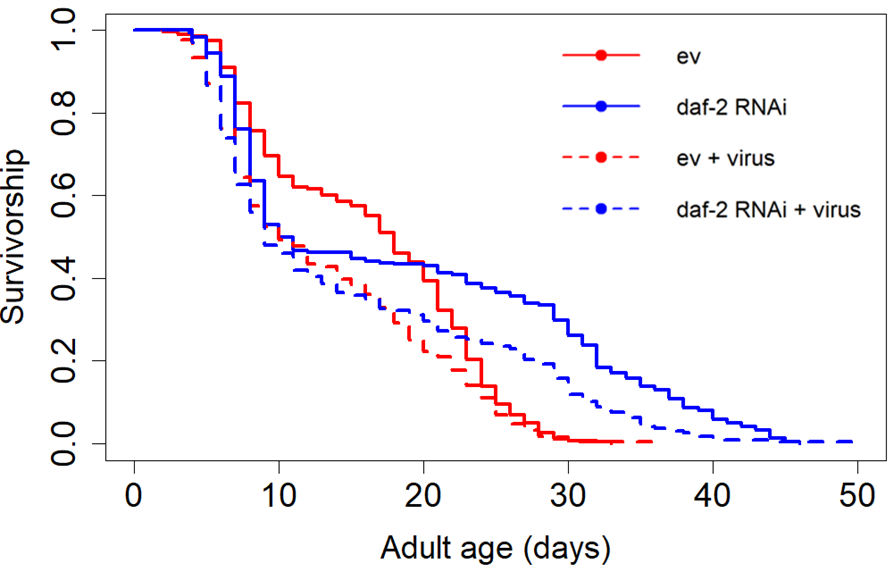
**

**Figure S2. Reduced insulin/IGF-1 signalling via *daf-2* RNAi in adulthood increases survival under Orsay virus infection in *C. elegans*.** Matricide was classed as death, age-specific survival data from four experimental blocks. The proportion of total individuals per treatment that died due to matricide was ev: 98/286, *daf-2* RNAi: 147/291, ev + virus: 133/308 and *daf-2* RNAi + virus: 159/310. Matricide occurred between day 2 and day 17 of adulthood, with 75% of matricides by day 9 and 50% between day 6 and day 9. *C. elegans* nematodes (JU2572 strain) were infected with Orsay virus (potent JUv2572 isolate) from egg stage, or eggs were bleached to remove viral infection. All nematodes were maintained on empty vector (ev) *E. coli* bacteria during development and then either *daf-2* RNAi or ev from late-L4 onwards.


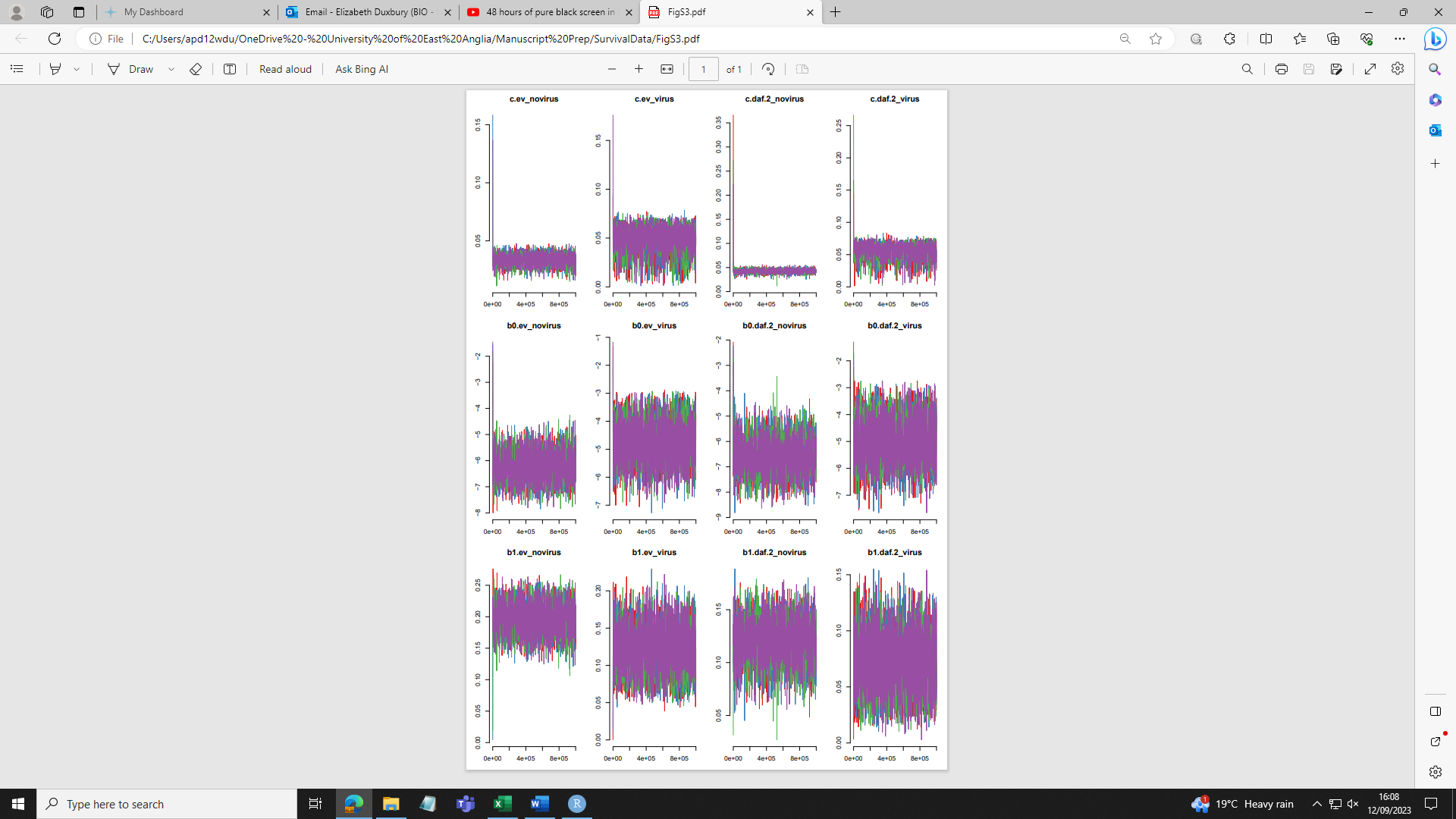
**Figure S3. BaSTA traces showing convergence for all parameter estimates across all experimental treatments, from the best-fitting Gompertz Makeham mortality model ran for 1000 000 iterations.**

**Supplementary Tables**

**Table S1. Cox proportional hazards mixed-effects model outputs for age-specific survival analyses.**

| **Variable** | **Coeff** | **z** | **p** | **Variance** |
| --- | --- | --- | --- | --- |
| Model 1: Full model for survival with matricide censored | | | | |
| Virus | 0.661 | 4.400 | **< 0.001** |  |
| RNAi | 1.708 | 10.930 | **< 0.001** |  |
| Virus x RNAi | -0.440 | -2.220 | **0.027** |  |
| PlateID |  |  |  | 0.0538 |
| Block |  |  |  | 0.0407 |
| Model 2: Survival for *daf-2* RNAi treated individuals with matricide censored | | | | |
| Virus | 1.064 | 5.970 | **< 0.001** |  |
| PlateID |  |  |  | 0.163 |
| Block |  |  |  | 0.0928 |
| Model 3: Survival for daf-2 RNAi + virus versus uninfected controls with matricide censored | | | | |
| RNAi | 1.003 | 6.930 | **< 0.001** |  |
| PlateID |  |  |  | < 0.001 |
| Block |  |  |  | 0.0448 |
| Model 4: Full model for survival with matricide treated as death | | | | |
| Virus | 0.913 | 2.930 | **0.00340** |  |
| RNAi | -0.127 | -0.480 | 0.630 |  |
| Virus x RNAi | 0.318 | 0.710 | 0.480 |  |
| PlateID |  |  |  | 4.708 |
| Block |  |  |  | 0.717 |
| Model 5: Full model for survival with matricide as dead and non-significant interaction removed | | | | |
| Virus | 1.060 | 4.530 | **p < 0.001** |  |
| RNAi | 0.060 | 0.390 | **p < 0.001** |  |
| PlateID |  |  |  | 4.716 |
| Block |  |  |  | 0.639 |

**Table S2. Parameter estimates for each treatment from the Gompertz Makeham best fitting model.** RNAi treatments were adulthood *daf-2* RNAi (“daf-2”) or empty vector (ev) and nematodes were either virally infected or not. Serial autocorrelation is shown. Convergence was reached for all parameters.

| **Parameter** | **Treatment** | **Estimate** | **SE** | **Lower**  **95%CI** | **Upper**  **95%CI** | **Serial Autocorr.** | **Update**  **Rate** |
| --- | --- | --- | --- | --- | --- | --- | --- |
| c | ev | 0.037 | 0.005 | 0.023 | 0.043 | <0.001 | 0.257 |
| c | ev + virus | 0.050 | 0.013 | 0.017 | 0.069 | 0.012 | 0.262 |
| c | daf-2 | 0.043 | 0.004 | 0.034 | 0.051 | 0.018 | 0.253 |
| c | daf-2 + virus | 0.057 | 0.011 | 0.029 | 0.073 | 0.011 | 0.250 |
| b0 | ev | -6.210 | 0.554 | -7.277 | -5.104 | 0.018 | 0.256 |
| b0 | ev + virus | -4.763 | 0.747 | -6.187 | -3.326 | <0.001 | 0.253 |
| b0 | daf-2 | -6.625 | 0.675 | -7.904 | -5.215 | 0.029 | 0.247 |
| b0 | daf-2 + virus | -4.979 | 0.845 | -6.560 | -3.350 | <0.001 | 0.262 |
| b1 | ev | 0.197 | 0.024 | 0.148 | 0.244 | <0.001 | 0.249 |
| b1 | ev + virus | 0.123 | 0.031 | 0.063 | 0.182 | 0.016 | 0.259 |
| b1 | daf-2 | 0.122 | 0.020 | 0.078 | 0.159 | 0.028 | 0.247 |
| b1 | daf-2 + virus | 0.073 | 0.027 | 0.023 | 0.124 | <0.001 | 0.248 |
| pi | pi.1 | 0.960 | 0.002 | 0.957 | 0.963 | 0.007 | 1.000 |

**Table S3. Kullback-Leibler divergence calibration (KLDC) values for all pairwise comparisons of treatments for each mortality parameter from Gompertz Makeham model.** RNAi treatments were adulthood *daf-2* RNAi (“daf-2”) or empty vector (ev) and nematodes were either virally infected or not. See Methods for details.

| **Treatment Comparison** | b0 | b1 | c |
| --- | --- | --- | --- |
| ev + virus vs ev | 0.957 | 0.985 | 0.931 |
| daf-2 vs ev | 0.619 | 0.998 | 0.929 |
| daf-2 + virus vs ev | 0.908 | 1.000 | 0.981 |
| daf-2 + virus vs daf-2 | 0.952 | 0.942 | 0.936 |
| daf-2 + virus vs ev + virus | 0.525 | 0.890 | 0.590 |
| daf-2 vs ev + virus | 0.983 | 0.583 | 0.890 |

**Table S4. Generalised linear mixed-effects model outputs for age-specific reproduction analyses.** Models were fitted using the glmmTMB package. The reference levels are no virus infection and *daf-2* RNAi. Significant effects are in bold. More details in the Methods. Model 2 had best fit with lowest AIC (dAIC = 188.9).

| **Variable** | **Estimate +/- SE** | **z** | **p** | **Variance** |
| --- | --- | --- | --- | --- |
| Model 1: Full Generalised Poisson model | | | | |
| Intercept | 3.87 +/- 0.31 | 12.680 | **< 0.001** |  |
| Virus | -1.24 +/- 0.15 | -8.355 | **< 0.001** |  |
| RNAi | -0.18 +/- 0.15 | -1.247 | 0.212 |  |
| Age | 0.48 +/- 0.09 | 5.448 | **< 0.001** |  |
| Age^2^ | -0.20 +/- 0.02 | -12.214 | **< 0.001** |  |
| Virus x RNAi | -0.04 +/- 0.21 | -0.211 | 0.833 |  |
| RNAi x Age | 0.18 +/- 0.13 | 1.412 | 0.158 |  |
| Virus Age | 0.32 +/- 0.12 | 2.645 | **0.008** |  |
| RNAi x Age^2^ | -0.04 +/- 0.02 | -1.598 | 0.110 |  |
| Virus x Age^2^ | 0.01 +/- 0.02 | 0.470 | 0.638 |  |
| RNAi x Virus x Age | 0.05 +/- 0.17 | 0.280 | 0.779 |  |
| RNAi x Virus x Age^2^ | -0.01 +/- 0.03 | -0.354 | 0.723 |  |
| PlateID |  |  |  | 0.178 |
| Block |  |  |  | 0.123 |
| Experimenter |  |  |  | 0.338 |
| Model 2: Full Zero-Inflated Generalised Poisson model (zi = ~Age + Age^2^) | | | | |
| Intercept | 3.76 +/- 0.33 | 11.514 | **< 0.001** |  |
| Virus | -1.16 +/- 0.15 | -7.718 | **< 0.001** |  |
| RNAi | -0.22 +/- 0.14 | -1.642 | 0.101 |  |
| Age | 0.62 +/- 0.08 | 7.311 | **< 0.001** |  |
| Age^2^ | -0.22 +/- 0.02 | -13.862 | **< 0.001** |  |
| Virus x RNAi | -0.08 +/- 0.22 | -0.380 | 0.704 |  |
| RNAi x Age | 0.22 +/- 0.12 | 1.865 | 0.062 |  |
| Virus x Age | 0.25 +/- 0.12 | 2.048 | **0.041** |  |
| RNAi x Age^2^ | -0.04 +/- 0.02 | -1.932 | 0.053 |  |
| Virus x Age^2^ | 0.02 +/- 0.02 | 0.904 | 0.366 |  |
| RNAi x Virus x Age | 0.05 +/- 0.17 | 0.310 | 0.757 |  |
| RNAi x Virus x Age^2^ | -0.01 +/-0.03 | -0.361 | 0.718 |  |
| zi model  Intercept  Age  Age^2^ | -10.40 +/- 0.76  4.14 +/- 0.40  -0.49 +/- 0.05 | -13.664  10.466  -9.268 | **< 0.001**  **< 0.001**  **< 0.001** |  |
| PlateID |  |  |  | 0.105 |
| Block |  |  |  | 0.399 |
| Experimenter |  |  |  | 0.612 |

**Table S5. Generalised linear model outputs for total reproduction analyses.** The reference levels are no virus infection and *daf-2* RNAi. Significant effects are in bold.

| **Variable** | **Estimate +/- SE** | **t** | **p** | **Variance** |
| --- | --- | --- | --- | --- |
| Intercept | 223 +/- 8.41 | 26.517 | **< 0.001** |  |
| Virus | -84.30 +/- 6.42 | -13.141 | **< 0.001** |  |
| RNAi | -1.47 +/- 6.61 | -0.222 | 0.825 |  |
| Virus x RNAi | -1.97 +/- 8.97 | -0.220 | 0.826 |  |
| Block |  |  |  | 231 |

**Table S6. Primer sequences.** Forward (fwd) and reverse (rev) sequences are listed in the 5’ to 3’ direction. Primers were designed based on MIQUE guidelines [1], and taken from [2] for *drh-1* and *cde-1,* [3] for OrV RNA-1 and [4] for *actin-3*.

| **Gene** | **Primer Sequences** |
| --- | --- |
| *drh-1*  (F15B10.2) | Fwd: CCTGACGGATGAGCAACAAG  Rev: CCACTGTATTCACTTCAATCAACTG |
| *cde-1*  (K10D2.3) | Fwd: CTGGAAGTAGGCGGAGAGGA  Rev: GAGGAGGAAGCCGAATCAGC |
| OrV RNA-1  (GW194 & GW195) | Fwd: ACCTCACAACTGCCATCTACA  Rev: GACGCTTCCAAGATTGGTATTGGT |
| *actin-3*  (T04C12.4) | Fwd: CCAAGAGAGGTATCCTTACCCTCAA  Rev: AAGCTCATTGTAGAAGGTGTGATGC |

**Table S7. Model comparison of ten age-specific mortality models run with Bayesian survival trajectory analysis (BaSTA).** Deviance Information Criteria (DIC) values were compared to select the best fitting model with lowest DIC. K indicates the effective number of parameters. All models tested (Gompertz, GO; Logistic, LO; Weibull, WE and Exponential, EX) with specified shapes, converged.

| **Model** | **Shape** | **K** | **DIC** | **∆DIC** | **Rank** |
| --- | --- | --- | --- | --- | --- |
| GO | Makeham | 13 | 13470 | 0 | 1 |
| LO | Makeham | 17 | 13487 | 17.1 | 2 |
| WE | Makeham | 13 | 13525 | 54.8 | 3 |
| GO | Bathtub | 21 | 13525 | 55.1 | 4 |
| GO | Simple | 9 | 13525 | 55.1 | 5 |
| WE | Bathtub | 21 | 13526 | 55.8 | 6 |
| LO | Bathtub | 25 | 13549 | 78.9 | 7 |
| WE | Simple | 9 | 13558 | 88.4 | 8 |
| LO | Simple | 13 | 13588 | 117.5 | 9 |
| EX | Simple | 5 | 13753 | 282.5 | 10 |

**Supplementary References**

1. Bustin SA, Benes V, Garson JA, Hellemans J, Huggett J et al. The MIQE Guidelines: minimum information for publication of quantitative real-time PCR experiments. Clinical Chemistry 2009; 55: 611-622.
2. Chauve L, Le Pen J, Hodge F, Todtenhaupt P, Biggins L et al. High-throughput quantitative RT-PCR in single and bulk *C. elegans* samples using nanofluidic technology. Journal of Visualised Experiments 2020; 159: e61132.
3. Felix M-A, Ashe A, Piffaretti J, Wu G, Nuez I, et al. Natural and experimental infection of *Caenorhabditis* nematodes by novel viruses related to Nodaviruses. PLoS Biology 2011; 9: e1000586.
4. Akay A, Di Domenico T, Suen KM, Nabih A, Parada GE et al. The helicase aquarius/EMB-4 is required to overcome intronic barriers to allow nuclear RNAi pathways to heritably silence transcription. Developmental Cell 2017; 42: 241-255.
